# Aromatic Amino Acid Decarboxylase Deficiency: The Added Value of Biochemistry

**DOI:** 10.3390/ijms22063146

**Published:** 2021-03-19

**Authors:** Riccardo Montioli, Carla Borri Voltattorni

**Affiliations:** Department of Neurosciences, Biomedicine and Movement Sciences, Section of Biological Chemistry, University of Verona, Strada Le Grazie, 8, 37134 Verona, Italy

**Keywords:** aromatic amino acid decarboxylase, dopa decarboxylase, AADC deficiency, pyridoxal 5′-phophate, pathogenic variants, rare disease

## Abstract

Aromatic amino acid decarboxylase (AADC) deficiency is a rare, autosomal recessive neurometabolic disorder caused by mutations in the *DDC* gene, leading to a deficit of AADC, a pyridoxal 5′-phosphate requiring enzyme that catalyzes the decarboxylation of L-Dopa and L-5-hydroxytryptophan in dopamine and serotonin, respectively. Although clinical and genetic studies have given the major contribution to the diagnosis and therapy of AADC deficiency, biochemical investigations have also helped the comprehension of this disorder at a molecular level. Here, we reported the steps leading to the elucidation of the functional and structural features of the enzyme that were useful to identify the different molecular defects caused by the mutations, either in homozygosis or in heterozygosis, associated with AADC deficiency. By revisiting the biochemical data available on the characterization of the pathogenic variants in the purified recombinant form, and interpreting them on the basis of the structure-function relationship of AADC, it was possible: (i) to define the enzymatic phenotype of patients harboring pathogenic mutations and at the same time to propose specific therapeutic managements, and (ii) to identify residues and/or regions of the enzyme relevant for catalysis and/or folding of AADC.

## 1. Introduction

Dopa or aromatic amino acid decarboxylase (DDC or AADC) (E.C.4.1.1.28) is a homodimeric pyridoxal 5′-phosphate (PLP) enzyme responsible for the production of the neurotransmitters dopamine and serotonin. DDC belongs to the fold-type I superfamily, in particular to the subgroup II of α-decarboxylases. The malfunction of AADC causes a rare inborn neurometabolic disorder, named AADC deficiency (MIM#608643), which was first described by Hyland and Clinton in 1990 [1]. The most commonly reported symptoms of AADC deficiency include neurodevelopmental delay, hypotonia, oculogyric crises, and a complex movement disorder with autonomic features [2,3,4]. The cerebrospinal fluid neurotransmitters profile, based on clinical symptoms, is highly indicative for the diagnosis of AADC deficiency [3]. Low levels of 5-hydroxyindoleacetic acid and homovanillic acid together with elevated concentrations of L-Dopa, L-5-hydroxytryptophan (L-5HTP), and 3-O-methylDopa are traditional diagnostic markers for AADC deficiency [3]. Both the quantification of plasma AADC enzyme activity and AADC enzyme gene sequencing contribute to confirming diagnosis. The most frequently used drugs are dopamine agonists, monoamine oxidase inhibitors, pyridoxal phosphate, pyridoxine, anticholinergic agents, folinic acid, and L-Dopa with or without carbidopa. However, responses to treatment are often not uniform and unsatisfying. Sometimes patients are treated with a mixture of many drugs, while only one or just a few drugs are used in other cases [3]. L-Dopa or dopamine agonists show an efficient action only towards a few patients, being instead inactive in the majority of cases [5,6]. In conclusion, therefore, it is very often quite difficult to foresee which might be a really good treatment. In order to improve the care for AADCD patients around the world, Wassenberg et al. [7] published a consensus guideline for the diagnosis and treatment of AADC deficiency. To date, 82 variants associated with AADC deficiency have been identified and catalogued among 123 patients. A recent review reports the current status of knowledge of AADC deficiency, with special emphasis on epidemiological, molecular genetic data, and novel gene therapy approaches to treatment [8]. As in all diseases related to the malfunction of an enzyme, it is therapeutically essential to understand the multiple mechanisms that relate the specific mutations to the pathology. In fact, missense mutations can cause functional (loss of catalytic activity) and/or structural (reduced expression level, protein misfolding or instability, aggregation propensity) defects. Therefore, the characteristics of the pathogenic variants and their comparison with those of the wild-type enzyme are essential to identify the molecular effects of each disease-causing mutation, thus identifying the enzymatic phenotype.

In this paper, our purpose was to highlight how important biochemical studies on the functional and structural features of AADC have been for the identification of the molecular defects of the variants associated with AADC deficiency either in homozygosis or in heterozygosis, as well as, as a consequence, to provide an experimental framework useful to suggest appropriate therapeutic treatments. On the other hand, the characterization of the purified recombinant form of several pathogenic variants allowed us to unravel the relevance of some residues in the catalytic mechanism of the enzyme.

### 1.1. Functional and Structural Features of AADC

Studies on the purification and preliminary characterization of AADC from pig kidney (pkDDC or pkAADC) [9,10,11,12] and rat liver (rlDDC or rlAADC) [13] were published about fifty years ago. Both enzymes have been cloned and expressed in *Escherichia coli* [14,15,16]. Thereafter, the human AADC (hDDC or hAADC) was also cloned, expressed in *Escherichia coli*, and characterized [17,18]. A great part of the information on the enzyme derives from studies of pkDDC and, even if at a lower level, from work on rlDDC. The complete amino acid sequence of pkDDC was reported in 1991 [19], the reaction specificity of the enzyme was widely investigated [20,21,22], and its X-ray structure in the holo form was solved in 2001 [23]. The substrate specificity, the steady state kinetic parameters, and the spectral properties of the enzyme itself or in the presence of substrates and substrate analogues, as well as the susceptibility to protease of pkDDC, rlDDC, and hDDC enzymes, have been defined and described [7,13,20,24]. Overall, the studies on the structural, catalytic, and inhibition properties of the enzyme indicated that the naturally occurring and recombinant mammalian AADC enzymes share structural and functional features. Here, we focused our attention on the enzyme characteristics useful to identify the defects caused by the mutations associated with AADC deficiency. The data reported concern either pkDDC or rlDDC and, if not specified, hDDC.

### 1.2. Kinetic Data and Reaction Specificity

**Figure 1:**
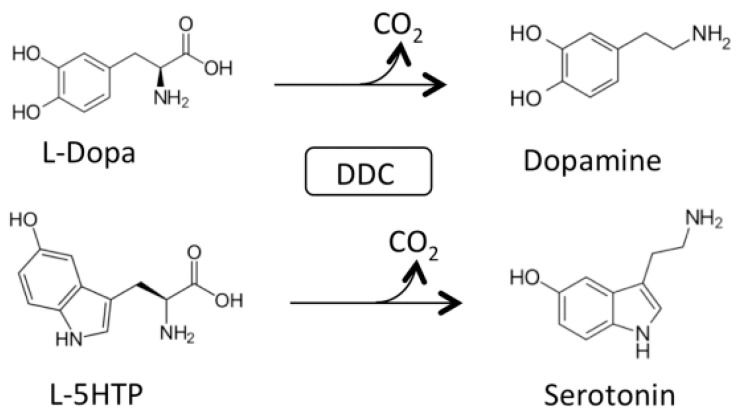
Main physiological reactions of Dopa decarboxylase (DDC). Decarboxylation of L-Dopa and L-5-HTP catalyzed by DDC.

**Figure 2:**
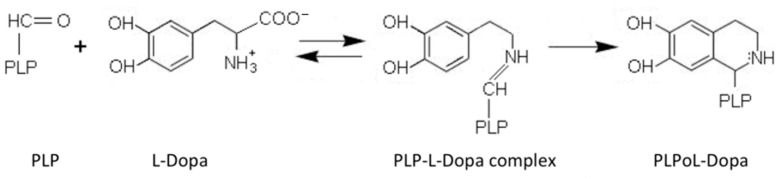
Pictet–Spengler reaction of pyridoxal 5′-phosphate (PLP). Scheme of the condensation reaction between PLP and L-Dopa generating the Pictet–Spengler adduct.

AADC catalyzes the following decarboxylation reactions (Figure 1):

AADC exhibits catalytic efficiency (*k*_cat_/K_m_) values of 70.6 and 20 mM^−1^min^−1^ for L-Dopa and L-5HTP, respectively, resulting from *k*_cat_ and K_m_ values of 7.6 s^−1^ and 0.11 mM for L-Dopa, and 1 s^−1^ and 0.05 mM for L-5HTP. The equilibrium dissociation constant of AADC for PLP (K_D(PLP)_) is of about 40 nM [18]. Along with the decarboxylation reaction, the enzyme catalyzes as a side reaction an abortive transamination with turnover numbers measured in seconds. Upon binding of D-dopa or D-5-HTP to the active site of DDC, half-transamination takes place concomitantly with a Pictet–Spengler reaction between D-aromatic amino acid and bound PLP, giving rise to a cyclic PLP adduct [21]. Abortive transamination proceeds through protonation of the quinonoid at the C4′ position producing the ketimine, which, after hydrolysis, releases an aldehyde and pyridoxamine 5′-phosphate. The Pictet–Spengler reaction is an organic reaction in which the α-amino group of the aromatic amino acid undergoes condensation with the aldehydic group of PLP followed by ring closure (Figure 2).

### 1.3. Spectroscopic Features of Internal and External Aldimines

The AADC-bound coenzyme gives rise to two absorbance bands at 420 and 335 nm associated with positive dichroic signals at the same wavelengths, which were attributed to a ketoenamine and an enolimine internal Schiff base, respectively [18]. The spectroscopic pH titration of the internal aldimine revealed that the 420 nm band decreased with pH, while the 335 nm band increased, and identified a single pK of ~7.2, possibly ascribable to an unidentified enzymatic residue governing the equilibrium between the low- and the high-pH forms of the internal aldimine [17]. The external aldimine observed in the presence of L-Dopa or L-5HTP was characterized by an immediate increase of the 420 nm absorbance band that was ascribed to the 4′-N-protonated external aldimine, followed by its decrease that paralleled the emergence of a 390 peak assigned to the 4′-N-unprotonated external aldimine [17]. Moreover, binding of L-5HTP to DDC resulted in the inversion of the 420 nm CD signal, i.e., in the disappearance of the original positive CD and its replacement by a negative CD shifted to 440 nm, and in the increase of the 335 nm dichroic band [18]. This strongly suggested that a change of the PLP microenvironment accompanied the conversion of the internal to the external aldimine.

### 1.4. Identification of Structural and/or Functional Residues in pkDDC and rlDDC Enzymes

Before the acquisition of the enzyme crystal structure, several attempts were performed to identify structural and/or functional residues of pkDDC and rlDDC enzymes. A series of systematic mutations of rlDDC residues have been carried out [25]. The residues were chosen since they appeared to be invariant or conservatively substituted on the basis of the alignment of the sequences of 13 group II amino acid decarboxylases. The catalytic activity of 18 mutant enzymes had been determined and some results and proposals are the following. Both K303A and K303R mutant pkDDC enzymes (Lys303 identified as the PLP-bound residue in pkDDC [26]) did not show any measurable activity, possibly because of the impairment of the transaldimination process between the Lys303-PLP Schiff base and the substrate (product)-PLP Schiff base. About 10 years later, evidence was provided that the active site lysine of pkDDC increases the coenzyme binding, accelerating the Schiff base formation and decay, and allowing a rapid product release [27]. D271A mutation abolishes the activity of rlDDC, whereas the mutation of Asp271 with Glu decreases by 1000-fold the *k*_cat_ value. These data, in good agreement with those concerning pkDDC [28], supported the view that the carboxylate group of Asp271 stabilizes the N-protonated form of the pyridine ring of the coenzyme, thus enhancing the decarboxylase activity. The finding that the decarboxylase activity was undetectable upon replacement of Arg355 with Ala, while it was not affected when Arg355 was substituted by Lys, could indicate that a positive charge is required at position 355 of rlDDC. In this regard, the authors were reminded that an Arg residue essential for the recognition of the α-carboxylate group of the substrate of pkDDC was found by chemical modification studies using phenylglyoxal [29]. Again, His192A did not show any detectable activity. It is likely that His192 participated in the catalytic reaction of DDC, considering also that modification of a single histidine residue with diethylpyrocarbonate resulted in the abolition of pkDDC activity [30].

The role of His192, Asp271, Asn300, and His302 residues of pkDDC, potential participants in catalysis because they belong to the common PLP-binding structural motif of group I, II, III decarboxylases and other PLP-enzymes, have been investigated [28]. The characterization of the H192Q, D271E, N300A, and H302Q artificial pkDDC mutants revealed that the H192Q, D271E, N300A, and H302Q mutations cause, even if at a different degree, an altered state of the coenzyme bound, a perturbation of the overall conformation of the enzyme, and a reduced decarboxylase activity accompanied by a half-transamination reaction. These data indicated that, although none of the mutated residues is essential for catalysis, they alter the nature of the catalysis possibly because of the loss of the integrity of the PLP cleft. The role of Cys111 in pkDDC had also been investigated [31]. This study originated from a previous work in which, by using the coenzyme affinity analog N-(bromoacetyl-1) pyridoxamine 5′-phosphate, it was demonstrated that Cys111 is specifically labeled in apopkDDC, suggesting that the residue lies at or near the active site [32]. With respect to the wild-type enzyme, the C111A and C111S pkDDC mutants displayed increased K_D(PLP)_ values, slower kinetics of reconstitution to holoenzyme, a decreased ability to anchor the external aldimine formed between D- Dopa and bound coenzyme, as well as structural changes. Overall, these results indicated that Cys111 is catalytically not essential, and supported the idea that the residue might be located at or near the active site and might be required for the maintenance of a proper active site conformation.

### 1.5. Susceptibility to Protease

The first and important step towards the understanding of the structure/function relationship operating in AADC was the finding that limited trypsin digestion of pkDDC yields two fragments of about 38 and 14 kDa produced by the cleavage of the Lys334–His335 peptide bond. These two fragments remain associated under native conditions [33]. The same cleavage occurs in the region around Arg334 of rlDDC, which is homologous to the region around Lys334 in pkDDC [34]. Protection studies against trypsin limited proteolysis indicated that, although the accessibility to trypsin of the cleavage site Lys334–His335 decreases significantly upon binding of L-aromatic amino acids, it does not change appreciably upon binding of D-aromatic amino acids. Moreover, the nicked enzymatic species upon reaction with L-aromatic amino acids (i) was able to form an external aldimine whose spectroscopic features were different from those of the native enzyme, (ii) gave rise to the formation of Pictet–Spengler L-aromatic amino acid adducts, and (iii) did not show decarboxylase activity. On the contrary, it retained a large percentage of the native transaminase activity toward D-aromatic amino acids accompanied by the formation of cyclic coenzyme-substrate adducts [21]. These data have suggested that upon binding of L- or D-aromatic amino acids, different conformational changes occur near residues at the tryptic cleavage site belonging to a flexible and exposed 11-residue loop (residues 328–339). The closure of this loop upon L-aromatic amino acid binding appeared to be essential for decarboxylase activity, and a catalytic role for Tyr332, located in the center of the loop, has been proposed. Tyr332 is predicted to function as a proton donor for the quinonoid intermediate following CO_2_ release [35]. Three distinct mobile catalytic loop conformations of DDC have been proposed: an “open” state adopted by the unliganded enzyme, a “closed” state (L-aromatic amino acid-induced) essential for decarboxylase activity, and a “half-open” or “semi-closed” state (D-aromatic amino acid-induced). The latter is characterized by a structure looser than that of the “closed” state leading to abortive transamination and the Pictet–Spengler reaction. It has been proposed that the uncleaved dynamic loop is essential for the transition open → closed conformation, while it is not critical for the “open” → “half-closed” conversion [21].

### 1.6. Crystal Structure of DDC in the Holo and Apo Form

**Figure 3:**
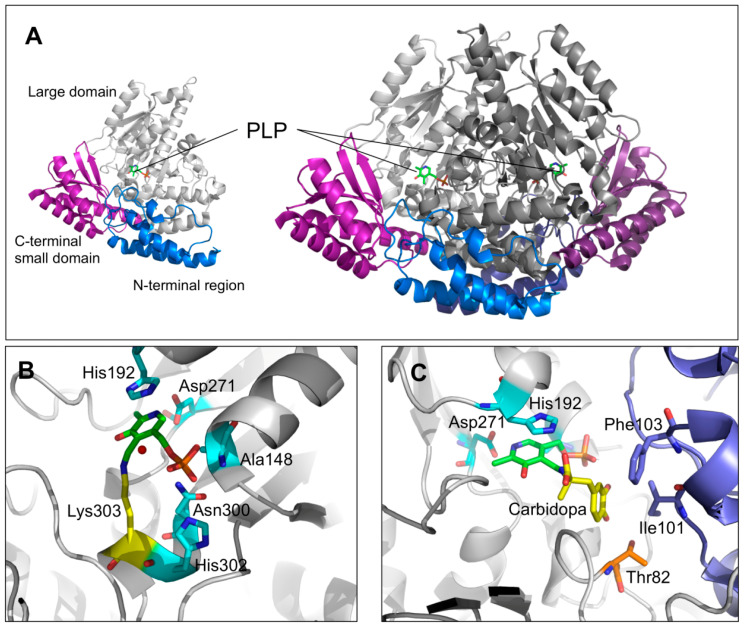
DDC structural features and active site architecture. (**A**) Ribbons represent the DDC monomeric and dimeric units. The N-terminal, large, and C-terminal domains are colored in blue, gray, and magenta, respectively. (**B**) Active site of unliganded holo-DDC or (**C**) in complex with the inhibitor carbidopa. PLP molecules are represented as green sticks, the PLP binding residues are represented as cyan sticks. The residues in the proper position to interact with the carbidopa catechol ring are highlighted. Image was rendered by PyMol software (Schrödinger).

The crystal structure of pkDDC in the holo form was solved at 2.6 Å [23]. The overall conformation of AADC consisted in a tightly associated dimer. Each subunit included (i) a large domain (86–360 residues) containing the PLP binding site and consisting of a central, seven-stranded mixed β-sheet surrounded by eight α-helices in a typical α/β fold, (ii) a C-terminal domain (361–480 residues) comprising a four-stranded antiparallel β-sheet packed against the face opposite to the large domain, and (iii) an N-terminal domain (1–85 residues), comprising two parallel helices linked by an extended strand, packing on the top of the large domain (Figure 3A). The short stretch of 11 amino acids (residues 328–339), representing a mobile loop important for the catalysis [21,33], was not ordered enough to be built in the crystal structure of the enzyme. PLP, covalently bound through a Schiff base to the active site Lys 303 (internal aldimine), was located at the subunit interface and interacted with residues of the dimer subunits. Besides the interaction with Lys303, the PLP molecule was non-covalently bound to the active site by a base stacking interaction between the His192 side chain and the PLP pyridine ring, a salt bridge between the Asp271 and the N1 of PLP as well as by several possible hydrogen bonds connecting the phosphate group of the coenzyme with three residues (Ala148, Asn300, and His302) and a water molecule (Figure 3B). The crystal structure of pkDDC complexed with the anti-Parkinson drug carbidopa was also solved. The binding of carbidopa, occurring through a hydrazone linkage with PLP and its hydrazinic group, being the carboxylate moiety approximately orthogonal to the PLP ring, mimicked the external aldimine enzyme–substrate intermediate, even if the carbidopa–PLP adduct, compared to the Schiff base complex with L-DOPA, contained an additional N–N bond. The catechol ring of carbidopa, deeply buried in the active site cleft, was in contact with the active site residues Ile101 and Phe303 provided by the adjacent monomer, while the 3′ and 4′ catechol hydroxyl groups were hydrogen bonded with the coenzyme phosphate and the hydroxyl groups of Thr82, respectively (Figure 3C) [23].

**Figure 4:**
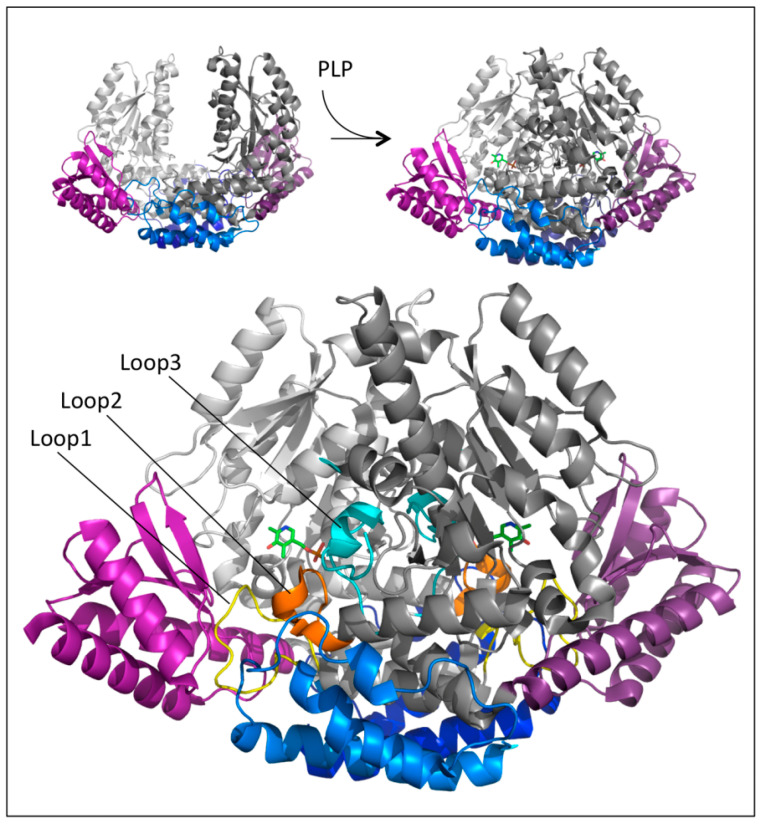
Structural regions involved in the apo-to-holo transition of DDC. Global conformational change accompanying the transition from the apo to the holo form of DDC. The N-terminal, large, and C-terminal domains are colored blue, gray, and magenta, respectively. The two monomers are distinguished by dark and light colors. The PLP molecules are represented as green sticks and loops 1, 2, and 3 are highlighted in yellow, orange, and cyan, respectively. Image was rendered by PyMol software (Schrödinger).

An unexpected and relevant finding was that the crystal structure of human apoDDC was very different from that of the pkDDC in the holo form [36]. The comparison between these two structures was plausible considering that hDDC is the close orthologue of pkDDC (89% sequence identity). Indeed, the resolution of the apo revealed that the structure appeared as an open bivalve shell in which the active site and the hydrophobic regions were partly exposed to the solvent, and the contact region between monomers comprised only the N-domains helices, which acted as the hinge. This crystal structure together with those of three alternative conformations of the active site at different PLP saturations allowed us to suggest that the PLP binding to the apoenzyme triggered an initial conformational change at the level of the active site, yielding the rearrangement of loop1 (residues 66 to 84) transmitted to loop2 (residues 100 to 110) and then to loop3 (residues 323–357), with both loops 2 and 3 belonging to the adjacent subunit (Figure 4).

### 1.7. Effects of Pathogenic Mutations

The solved crystal structures of DDC in the holo and apo forms provided structural scaffolds to explain the effects of some pathogenic mutations. Quite interesting was the presence of four mobile loops in DDC since their rearrangements were crucial to catalysis. In particular, loop1 underwent conformational changes upon PLP binding, giving rise to a correct apo → holo transition. These conformational changes were transmitted to loops 2 and 3 of the adjacent monomer, these loops becoming structured only in the closed conformation (Figure 4). Again, the catalytic loop underwent conformational changes as a response to substrate binding, leading to a closed productive conformation that was characterized by distinct interactions between the catalytic loop and the active site. Thus, the proper function of DDC required dynamic structural elements scattered across the surface of the protein. These structural determinants were very useful to interpret the molecular defects of the pathogenic variants of residues belonging to or interacting with these loops. On the other hand, mutations of residues located outside these loops, i.e., the large, N-terminal, and C-terminal domains, were interpreted with difficulty since the structure-function relationships of these regions are unknown. However, the impact of the residues belonging to these regions on the folding and catalytic activity of DDC improved the comprehension of the role of some residues of the enzyme. The structural elements along with the resulting catalytic effects have been taken into consideration during the characterization of the pathogenic variants, as reported in Table 1.

## 2. hDDC Pathogenic Mutations in Homozygosis

### 2.1. Mutations of Residues Belonging to Loop1

The His70Y, His72Y, F77L, Y79C, and P81L pathogenic variants in their holo form exhibited a linear correlation between the functional (a remarkable drop of catalytic activity) and the structural (a significant alteration of the tertiary structure) effects. These variants were also characterized, even if to different degrees, by an alteration of the chiral environment of PLP, an increase of the K_D(PLP)_ value, and changes of the CD features of the external aldimine. Instead, no significant effect has been observed in the tertiary structure of their apo form [37]. This structure–function relationship has also been observed for the T69M mutation as well as for the R447H, R453C, and R462P mutations, even if their impact is less pronounced. Interestingly, the Thr69, His70, His72, F77, Tyr79, and Pro81 residues map to loop1, and the Arg447, R453, and Arg462 residues interact with loop1 residues Phe80, Ser84, and Tyr75, respectively. These data allowed us to (i) substantiate the essential role of loop1 in the conversion from the open apo conformation to the closed holo form, and (ii) indicate that the molecular bases underlying the pathogenicity of these affected residues rely on the inability of DDC to achieve its catalytic active holo form. Thus, T69M, His70Y, His72Y, F77L, Y79C, and P81L, as well as the R447H, R453C, and R462P, can be regarded as catalytic and folding mutations (Table 2). In addition, these data strengthened the idea that the structural rearrangement of loop1 and the enzyme catalytic activity are highly interconnected.

### 2.2. Mutations of Residues Belonging to Loop2

The interfacial Ala110 residue is the last residue of the C-terminal of loop2, while Gly102 is a residue located in the proximity of the N-terminal end of loop2. The A110E variant displayed a consistent alteration of its tertiary structure and such a slow decarboxylation reaction that it became impossible to measure the kinetic parameters. Indeed, the initial velocity of decarboxylation was about 7.6 × 10^4^ times lower than that of the wild-type enzyme under the same experimental conditions [38]. It should be noted that when Ala in position 110 was substituted with Gln, the catalytic efficiency resulted in a ~17,000-fold decrease (resulting from a ~500-fold reduction of the *k*_cat_ value and a ~32-fold increase of the K_m_ value [37]), and the tertiary structure was consistently altered. On the other hand, the G102S variant showed no significant conformational changes, and K_m_ and *k*_cat_ values were ~11-fold higher and ~6-fold lower, respectively, than those of the wild-type enzyme [18]. Nevertheless, the A110E and G102S variants shared several defects consisting of an altered coenzyme microenvironment and of an incorrect orientation and/or anchoring of the external aldimines, as suggested by their CD features, the lack of protection upon substrate binding against limited trypsin proteolysis, and the occurrence of the Pictet–Spengler reaction accompanying the decarboxylation reaction. A similar altered reaction specificity has also been observed in the nicked enzyme [21]. However, the value of the partition ratio between the initial velocity of dopamine generation and cyclic adduct formation was for the G102S variant ~59,000-fold higher than that for the A110E variant. This could explain the different reduction in the *k*_cat_ values of these variants. The substitution of Gly102 with Ser could cause the displacement of Phe103, while the replacement of Ala110 with Glu could exert some effects on the position of Phe103 and Ile101. Both these residues are located at the active site and are involved in the interaction with the catechol ring of carbidopa (see above). These facts explain the increase of the K_m_ values of the G102 variant and the effects on the active site architecture of both variants. There was no evidence that the G102S and A110E mutations impaired in a macroscopic way the correct apo → holo conversion. Rather, they seemed to prevent upon substrate binding the achievement of the closed conformation productive for decarboxylation, in which the Pictet–Spengler reaction did not occur (Table 2). It is likely that binding of substrates could establish an equilibrium between a mixture of the half-open and closed conformation, and that the equilibrium position could shift toward the half-open state more for the A110E than for the G102S. However, further studies encompassing characterization of mutants of residues located on the middle of loop2 are needed to better clarify the role of this loop on the structural and functional features of DDC.

### 2.3. Mutations of Residues Belonging to Loop3

The R347Q and R347G variants, involving a residue mapping to loop3, can be classified as catalytic mutations since, albeit no conformational alteration was observed, they exhibited a remarkable decrease of the catalytic activity [39,40]. The combination of bioinformatics analyses with the characterization of the artificial F103L, D345A, and R347K variants was useful not only to give an explanation of the molecular basis underlying the pathogenicity of mutations of the Arg347 residue, but also for the identification of new residues important for catalysis. Indeed, a set of hydrogen bonds engaged by Arg347 with both Asp 345 and Leu333 (the latter residue belonging to the catalytic loop) were relevant for the decarboxylase activity since they represented structural determinants imposing important constrains on the alignment of the catalytic groups at the active site of DDC upon substrate binding [40]. These data pointed out that a proper contact between loop3 and the catalytic loop was a prerequisite for the achievement of a productive closed conformation. Like the mutations of the Arg347 residue, the L353P mutation, involving a residue located at the dimer interface, caused only a slight alteration of the enzyme tertiary structure, and a dramatic drop in decarboxylase activity, the generation of PLP-L-Dopa cyclic adduct during the reaction of L-Dopa, and the susceptibility of the L353P variant to tryptic proteolysis in the presence of a substrate analogue, in other words, a behavior similar to that observed for the A110E variant. The fact that Leu353 was at a proper distance to interact with Phe103, a critical residue for the active site conformation, and also with Arg347, a key catalytic residue, strongly suggests that the closing of the active site cleft of the L353P variant upon substrate binding did not occur. Like R347Q and R347G, the L353P can be considered a catalytic mutation (Table 2). It can be inferred that the mispositioning of the external aldimine at the active site of the R347G/Q and L353P variants is responsible for the catalytic defect. These data, together with the fact that loop3 comprised the catalytic loop, strongly suggest indeed a catalytic role for loop3.

### 2.4. Mutations Belonging to the Large, N-Terminal, and C-Terminal Domains (Outside the Loops)

Before the resolution of the crystal structure of holoDDC, it was only found that the artificial mutations concerning residues His192, Asn300, His302, Asp271 [28], and Cys111 [31] induced a perturbation of the active site geometry, leading to a decreased catalytic activity. This was in line with the location of these residues in the X-ray structure. In fact, His192 and Asp271 were directly involved in the binding of PLP at the active site, through a base stacking interaction and a salt bridge, respectively, while both Asn300 and His302 were in the proper position to contact the PLP phosphate group through a hydrogen bond (Figure 3B). Thus, it is not surprising that mutations of these residues affect the enzyme activity. On the other hand, Cys111 is located on the edge of loop2 and does not belong to the active site cleft. However, due to the knowledge acquired about the effects of loop2 alteration, we may suggest that, as observed for the neighbor Ala110, the perturbation of the C-terminal region of loop2 can heavily affect the active site [37,38].

Unlike the case of the catalytic loop and loops 1, 2, and 3, the understanding of the structure–function relationships of the large, N-terminal, and C-terminal domains is poor, and only some speculations can be advanced about the effects caused by the mutated pathogenic residues belonging to these regions. Starting from the large domain, it was noticed that the G123R and F309L mutations caused structural (change in the near-UV CD signal) and functional (change in the catalytic efficiency and in the spectroscopic features of the external aldimine) alterations similar, but less pronounced, to those observed for the mutated residues mapping to loop1 (Table 2) [37]. It can be suggested that these mutations might indirectly affect both the active site and loop residues. On the other hand, the P210L, L222P, F237S, S250F, W267R, R285W, and E283A variants displayed a low expression level in *Escherichia coli* (10–20% that of the wild-type enzyme) [38]. Moreover, except for the L222P and F237S variants for which no data on catalytic activity are available, they showed a modest decrease of their catalytic efficiency [38]. Thus, these variants had a higher folding defect than a catalytic one (Table 2). Pathogenic mutations prevented the correct folding of a protein leading to various downstream effects including aggregation and increased degradation. In this regard, it must be noticed that (i) the E328A variant in the apo form was prone to aggregate [38], and (ii) the S250F variant expressed in mammalian cells underwent degradation by the proteasomal pathway, which led to the accumulation of denatured and/or partially denatured forms of the variant prone to proteolytic degradation [41]. It is reasonable to advance the hypothesis that Leu 222, Phe237, Glu328, and Ser250 lay on a region outside loops 1, 2, and 3 of the large domain (Figure 3) which is relevant for the global folding process of the enzyme. This is corroborated by the low expression level and the modest decrease of the catalytic activity of the R160W variant (pathogenic only in the heterozygosis). These effects concerning a residue located at the dimer interface are possibly related to a folding defect in the dimerization step [40].

Gly96 is a residue at the monomer–monomer interface, and a high impact of this mutation on dimer stabilization has been predicted by measuring the ΔΔG of monomer–monomer interaction [38]. Nevertheless, the expression level of the G96R variant was unchanged and its tertiary structure was only slightly changed as compared to the wild-type enzyme. On the other hand, a ~32-fold decrease of the K_m_ value and a ~5-fold increase of the K_D(PLP)_ value were observed for this variant. It can be assumed that, although the G96R variant showed both structural and functional alterations, the change in the L-Dopa and in the PLP binding affinity could be the main molecular defects of the variant.

Instead, it is hard to explain the molecular basis of the pathogenicity of the F251S variant. Phe251 is located near the protein surface and participates in a hydrophobic network involving residues of the large and C-terminal domains. The F251S mutation did not cause significant changes in the catalytic and structural features of the enzyme [38]. Therefore, the molecular basis underlying the pathogenicity of F251S mutation needs further investigation.

As for the N-terminal domain, to date, three pathogenic variants, L38P, P47H, and V60A, have been identified. Although both the L38P and P47H mutations, each one of them involving residues far from the active site, induced a consistent similar impact on the tertiary structure of the enzyme, the substitution of Leu38 with Pro gave rise to a dramatic drop of the decarboxylase activity (~3800-fold lower that of the wild-type enzyme), while the substitution of Pro47 with His caused a small reduction (~4.5-fold) of the catalytic activity and a reduced expression level of the variant (Table 2) [37]. We may reasonably suggest that these mutated residues were not involved in the apo → holo conversion and that the types of amino acid replacement could be more responsible for the observed effects than their location and contacts with other residues. The functional and structural properties of the V60A variant are comparable to those of the variants of residues mapping to loop 1. In fact, remarkable changes have been observed in the near-UV CD signal, in the *k*_cat_ value, in the visible absorbance and CD spectra, and in the K_D(PLP)_ value of this variant with respect to the wild-type enzyme (Table 2) [38]. It is likely that the V60A substitution could compromise indirectly the hydrophobic contacts involved in the conformation of loops 1 and 2 and their rearrangements during the apo → holo transition. Mutations of the C-terminal domain associated with AADC deficiency identified so far are L408I, R412W, R447H, R462P, and R453C. The molecular basis of the pathogenicity of the latter three mutations has been highlighted above. Although both the L408I and R412W variants displayed a ~10-fold decreased decarboxylase activity, they showed, even if to a different extent, a change in their tertiary structure, and in the PLP binding mode and affinity [37]. Thus, both these mutations can be considered catalytic and folding mutations (Table 2), even if the structural bases of their effects are not clear. Considering the low expression level of the R412W variant in “*E. coli*”, it is possible that the drastic Arg → Trp substitution at position 412 could affect the folding pathway of the variant.

### 2.5. Classification of Pathogenic Mutations in Homozygosis

The determination of the crystal structure of DDC in the holo and apo form in combination with kinetic, spectroscopic, and bioinformatics studies of expressed purified recombinant forms of pathogenic variants has proven to be valuable in the interpretation of many mutations that cause AADC deficiency. The mutations associated with AADC deficiency can be grouped into three categories based on the distinct structural and functional effects of the mutations in each category: (1) Folding and catalytic, (2) catalytic, and (3) folding mutations (Table 2). Folding and catalytic mutations mostly concern residues mapping to or contacting loop1, but also some residues belonging to the large domain and indirectly interacting with loop1, residues mapping to loop2, as well as residues belonging to the N-terminal domain. In contrast, mere catalytic mutations concern residues belonging to loop3. Nonetheless, it should be noted that both the pathogenic variants of residues of loops 2 and 3 share the same catalytic defect, which consists in an incorrect location of the external aldimine at their active site. This is in agreement with the fact that the mutated residue(s) contact residues at the active site and/or residue(s) belonging to the catalytic loop. Folding mutations mainly involve residues located in a region of the large domain. Nonetheless, the molecular basis of the folding defect has been analyzed for only the S250F mutation.

## 3. hDDC Pathogenic Mutations in Heterozygosis

Another example of the relevant progress in understanding the pathogenicity of AADC deficiency provided by biochemical studies is the ability to define the enzymatic phenotype of patients bearing mutations in heterozygosis. Indeed, the conventional expression system does not allow us to define the impact of two mutations in heterozygosis since only a single mutant allele is expressed, thus producing a homoallelic protein. A pool of monomers, each one having a different mutation, together with heterodimers in which one monomer contains one mutation and the second monomer contains the other mutation should be virtually present in the compound heterozygous patients. Thus, their enzymatic phenotype depends not only on the effects due to the single mutations, but also on the effect that each single mutation has in the hybrid protein. Interallelic complementation (IC) effects could derive from heterodimeric species with functional and/or structural properties different from the average of those of the parental homodimers. The IC could lead to a less severe phenotype (positive IC) or a more severe one (negative IC) than those of the homozygous counterparts. The pathogenic mutations in heterozygosis considered here are: K347Q/R358H, K347Q/R160W, and C410G/A91V. In order to define the enzymatic phenotype of the patients harboring these mutations it was necessary to isolate, purify, and characterize the R347Q, R358H, R160W, C410G, and A91V homodimeric species as well as the K347/R358H, K347Q/R160W, and C410G/A91V heterodimers [40,42]. By using the dual-vector expression strategy, the heterodimers were constructed with the exception of the K347Q/R160W, which presented a very low expression level and an undetectable decarboxylase activity. As for the enzymatic phenotype of the R347Q/R358H mutation in heterozygosis, the data concerning the functional and structural features of the R347Q and the R358H homodimers as well as those of the R347Q/H358H heterodimer indicated that (i) the R347Q mutation was only defective in catalysis, while the R358H mutation induced both catalytic and structural defects, and (ii) the R347Q and R358H alleles complemented in a positive manner [40]. This could imply that compound heterozygous patients harboring the R347Q and R358H mutations could have an enzymatic phenotype milder than that of homozygous patients bearing the R347Q or R358H mutations. The molecular aspects of the C410G/A91V heterozygous mutations were also identified. The C410G mutation caused significant structural and functional effects, while the A91V mutation induced remarkable effects on both tertiary structure and catalysis, as well as an altered perturbation of the active site, which could be responsible for the occurrence of the Pictet–Spengler reaction during the reaction of the A91V homodimer with L-Dopa. It was also noticed that the C410G/A91V heterodimer displayed a *k*_cat_ ~ 2.6-fold lower than that predicted by averaging the *k*_cat_ values of the dimeric counterparts [42]. This suggests that the interplay between the C410G and A91V alleles might occur in a negative manner. Overall, these biochemical studies enriched the phenotypic spectrum of AADC deficiency.

## 4. Therapeutic Strategy

Understanding the molecular defects of pathogenic mutations is a pivot to progress in pathogenesis since it allows for the classification of the mutations in categories, thus suggesting specific therapeutic approaches. To date, treatment of AADC deficiency is supportive, even having only marginal effects on some symptoms. As already reported [2], the most common first-line treatments are bromocriptine, a dopamine receptor agonist, and pyridoxine. Other medications used in managing affected patients are monoamine oxidase inhibitors such as selegiline, pergolide, tranylcypromine, trihexyphenidyl, L-Dopa, and folinic acid. In many cases, the treatment consists in a combined therapy with pyridoxine, dopamine agonists, and monoamine oxidase inhibitors, which makes it difficult to attribute specific benefits or side effects to a single drug. Nonetheless, gene therapy is a promising experimental approach to AADC deficiency treatment, where the *DCC* gene may be transferred directly into patients’ cells to stabilize the expression of the AADC protein. On the basis of the above classification of pathogenic mutations, it can be suggested that a specific therapeutic management for AADC deficiency patients could consist of (i) administration of dopamine agonists and/or monoamine oxidase inhibitors that may be reasonable for patients harboring catalytic and folding mutations, (ii) administration of dopamine agonists that might be useful for patients bearing catalytic mutations, and (iii) administration of pyridoxine (which could act as a pharmacological chaperone) that may be very useful in the therapy of patients bearing folding mutations. L-Dopa and/or pyridoxine could be added to the therapeutic protocol for AADC-deficient patients carrying mutations causing high K_m_ and/or K_D(PLP)_ values.

## 5. Conclusions

The biochemical history of AADC deficiency stems from the understanding of the functional and structural properties of DDC. The resolution of the crystal structure of the enzyme in the apo and holo form, together with kinetic and spectroscopic features, represents an important frame in the molecular characterization of the variants associated with AADC deficiency. Thereafter, molecular biological and biochemical studies, devoted to construction, expression, purification, and characterization of many pathogenic variants in homozygosis and heterozygosis, have been essential to identify the molecular defects of each disease-causing mutation. The comparison of the functional and structural properties of the variants with those of the wild-type enzyme allowed us to list the mutations into three different groups, which made it possible to define the enzymatic phenotype of patients bearing these mutations. As a consequence, a differentiated therapeutic strategy was proposed for each group. These studies, however, have some limitations, since it is impossible to define an enzymatic-clinical phenotype correlation considering the poor number of patients carrying the examined pathogenic mutations. Nonetheless, an aspect of these studies that must be emphasized is that they unravel new residues and/or regions of the enzyme critical for catalysis and/or folding of DDC, thus improving the comprehension of its structure–function relationship.

## Figures and Tables

**Table 1:** Technique and readout.

Technique	Readout
Visual inspection of the X-ray structure and computational analyses	Loss of the interaction(s) of the mutated residue with other residues
Kinetic parameters for L-Dopa	Change in the *k*_cat_ and/or K_m_ values
Visible absorbance and CD spectra and K_D(PLP)_ value	Change in the PLP binding mode and affinity
Near-UV CD spectra, intrinsic, and ANS emission spectra	Change in the tertiary structure
Absorbance and CD spectra of the variant in the presence of L-Dopa or L-5HTP and time-dependence of tryptic cleavage in the presence of a substrate analogue	Change in the conformation of external aldimine
Identification of the reaction products	Change in the reaction specificity
Western blotting	Change in the expression level

**Table 2:** Classification of pathogenic mutations in homozygosis.

Catalytic and Folding Mutations	Catalytic Mutations	Folding Mutations
T69M (loop1)	R34Q (loop3)	P201L (large domain)
H70Y (loop1)	R347G (loop3)	L222P (large domain)
F77L (loop1)	L353P (loop3)	P237S (large domain)
Y79C (loop1)		S250F (large domain)
R447H (C-term domain)		R285W (large domain)
R453C (C-term domain)		E328A (large domain)
R462P (C-term domain)		P47H (N-term domain)
L408I (C-term domain)		
R412W (C-term domain)		
G123R (large domain)		
F309L (large domain)		
V60A (N-term domain)		
L38P (N-term domain)		
G102S (loop2)		
A110E (loop2)		

## Data Availability

All data reported are available on PubMed database (https://pubmed.ncbi.nlm.nih.gov/) (accessed on 18 March 2021).

## References

[1] Hyland K., Clayton P.T. (1990). Aromatic amino acid decarboxylase deficiency in twins. J. Inherit. Metab. Dis..

[2] Manegold C., Hoffmann G.F., Degen I., Ikonomidou H., Knust A., Laaß M.W., Pritsch M., Wilichowski E., Hörster F. (2009). Aromatic l-amino acid decarboxylase deficiency: Clinical features, drug therapy and follow-up. J. Inherit. Metab. Dis..

[3] Brun L., Ngu L.H., Keng W.T., Ch’Ng G.S., Choy Y.S., Hwu W.L., Lee W.T., Willemsen M.A.A.P., Verbeek M.M., Wassenberg T. (2010). Clinical and biochemical features of aromatic L-amino acid decarboxylase deficiency. Neurology.

[4] Pearson T.S., Gilbert L., Opladen T., Garcia-Cazorla A., Mastrangelo M., Leuzzi V., Tay S.K.H., Sykut-Cegielska J., Pons R., Mercimek-Andrews S. (2020). AADC deficiency from infancy to adulthood: Symptoms and developmental outcome in an international cohort of 63 pa-tients. J. Inherit. Metab. Dis..

[5] Chang Y.T., Sharma R., Marsh J.L., McPherson J.D., Bedell J.A., Knust A., Bräutigam C., Hoffmann G.F., Hyland K. (2004). Levodopa-responsive aromaticL-amino acid decarboxylase deficiency. Ann. Neurol..

[6] Leuzzi V., Mastrangelo M., Polizzi A., Artiola C., Van Kuilenburg A.B.P., Carducci C., Ruggieri M., Barone R., Tavazzi B., Abeling N.G.G.M. (2014). Report of Two Never Treated Adult Sisters with Aromatic l-Amino Acid Decarboxylase Deficiency: A Portrait of the Natural History of the Disease or an Expanding Phenotype?. JIMD Rep..

[7] Wassenberg T., Molero-Luis M., Jeltsch K., Hoffmann G.F., Assmann B., Blau N., Garcia-Cazorla A., Artuch R., Pons R., Pearson T.S. (2017). Consensus guideline for the diagnosis and treatment of aromatic l-amino acid decarboxylase (AADC) defi-ciency. Orphanet J. Rare Dis..

[8] Himmelreich N., Montioli R., Bertoldi M., Carducci C., Leuzzi V., Gemperle C., Berner T., Hyland K., Thöny B., Hoffmann G.F. (2019). Aromatic amino acid decarboxylase deficiency: Molecular and metabolic basis and therapeutic outlook. Mol. Genet. Metab..

[9] Voltattorni C.B., Minelli A., Vecchini P., Fiori A., Turano C. (1979). Purification and characterization of 3,4-dihydroxyphenylalanine decarboxyase from pig kidney. Eur. J. Biochem..

[10] Christenson J.G., Dairman W., Udenfriend S. (1970). Preparation and properties of a homogeneous aromatic L-amino acid decar-boxylase from hog kidney. Arch. Biochem. Biophys..

[11] Lancaster G.A., Sourkes T.L. (1972). Purification and Properties of Hog-Kidney 3,4-Dihydroxyphenylalanine Decarboxylase. Can. J. Biochem..

[12] Srinivasan K., Awapara J. (1978). Substrate specificity and other properties of DOPA decarboxylase from guinea pig kidneys. Biochim. Biophys. Acta Enzym..

[13] Dominici P., Tancini B., Barra D., Voltattorni C.B. (1987). Purification and characterization of rat-liver 3,4-dihydroxyphenylalanine decarboxylase. J. Biol. Inorg. Chem..

[14] Hayashi H., Mizuguchi H., Kagamiyama H. (1993). Rat liver aromatic L-amino acid decarboxylase: Spectroscopic and kinetic analysis of the coenzyme and reaction intermediates. Biochemistry.

[15] Moore P.S., Dominici P., Voltattorni C.B. (1996). Cloning and expression of pig kidney dopa decarboxylase: Comparison of the naturally occurring and recombinant enzymes. Biochem. J..

[16] Tanaka T., Horio Y., Taketoshi M., Imamura I., Ando-Yamamoto M., Kangawa K., Matsuo H., Kuroda M., Wada H. (1989). Molecular cloning and sequencing of a cDNA of rat dopa decarboxylase: Partial amino acid homologies with other enzymes synthesizing catecholamines. Proc. Natl. Acad. Sci. USA.

[17] Montioli R., Cellini B., Dindo M., Oppici E., Voltattorni C.B. (2013). Interaction of Human Dopa Decarboxylase with L-Dopa: Spectroscopic and Kinetic Studies as a Function of pH. BioMed Res. Int..

[18] Montioli R., Cellini B., Voltattorni C.B. (2011). Molecular insights into the pathogenicity of variants associated with the aromatic amino acid decarboxylase deficiency. J. Inherit. Metab. Dis..

[19] Maras B., Dominici P., Barra D., Bossa F., Voltattorni C.B. (1991). Pig kidney 3,4-dihydroxyphenylalanine (dopa) decarboxylase. Primary structure and relationships to other amino acid decarboxylases. J. Biol. Inorg. Chem..

[20] Voltattorni C.B., Minelli A., Dominici P. (1983). Interaction of aromatic amino acids in D and L forms with 3,4-dihydroxyphenylalanine decarboxylase from pig kidney. Biochemistry.

[21] Bertoldi M., Frigeri P., Paci M., Voltattorni C.B. (1999). Reaction Specificity of Native and Nicked 3,4-Dihydroxyphenylalanine Decarboxylase. J. Biol. Chem..

[22] O’Leary M.H., Baughn R.L. (1977). Decarboxylation-dependent transamination catalyzed by mammalian 3,4-dihydroxyphenylalanine decarboxylase. J. Biol. Chem..

[23] Burkhard P., Dominici P., Borri-Voltattorni C., Jansonius J.N., Malashkevich V.N. (2001). Structural insight into Parkinson’s disease treatment from drug-inhibited DOPA decarboxylase. Nat. Genet..

[24] Voltattorni C., Minelli A., Turano C. (1971). Spectral properties of the coenzyme bound to DOPA decarboxylase from pig kidney. FEBS Lett..

[25] Ishii S., Mizuguchi H., Nishino J., Hayashi H., Kagamiyama H. (1996). Functionally Important Residues of Aromatic L-Amino Acid Decarboxylase Probed by Sequence Alignment and Site-Directed Mutagenesis. J. Biochem..

[26] Bossa F., Martini F., Barra D., Voltattorni C., Minelli A., Turano C. (1977). The chymotryptic phosphopyridoxyl peptide of DOPA decarboxylase from pig kidney. Biochem. Biophys. Res. Commun..

[27] Bertoldi M., Voltattorni C.B. (2009). Multiple roles of the active site lysine of Dopa decarboxylase. Arch. Biochem. Biophys..

[28] Bertoldi M., Castellani S., Voltattorni C.B. (2001). Mutation of residues in the coenzyme binding pocket of Dopa decarboxylase. JBIC J. Biol. Inorg. Chem..

[29] Tancini B., Dominici P., Barra D., Voltattorni C.B. (1985). An essential arginine residue at the binding site of pig kidney 3,4-dihydroxyphenylalanine decarboxylase. Arch. Biochem. Biophys..

[30] Dominici P., Tancini B., Borri Voltattorni C. (1985). Chemical modification of pig kidney 3,4-dihydroxyphenylalanine decarboxylase with diethyl pyrocarbonate. Evidence for an essential histidyl residue. J. Biol. Chem..

[31] Dominici P., Moore P.S., Castellani S., Bertoldi M., Voltattorni C.B. (1997). Mutation of cysteine 111 in Dopa decarboxylase leads to active site perturbation. Protein Sci..

[32] Dominici P., Maras B., Mei G., Borri Voltattorni C. (1991). Affinity labeling of pig kidney 3,4-dihydroxyphenylalanine (Dopa) de-carboxylase with N-(bromoacetyl)pyridoxamine 5′-phosphate. Modification of an active-site cysteine. Eur. J. Biochem..

[33] Tancini B., Dominici P., Simmaco M., Schininà M.E., Barra D., Voltattorni C.B. (1988). Limited tryptic proteolysis of pig kidney 3,4-dihydroxyphenylalanine decarboxylase. Arch. Biochem. Biophys..

[34] Ishii S., Hayashi H., Okamoto A., Kagamiyama H. (1998). Aromatic L-amino acid decarboxylase: Conformational change in the flexible region around arg334 is required during the transaldimination process. Protein Sci..

[35] Bertoldi M., Gonsalvi M., Contestabile R., Voltattorni C.B. (2002). Mutation of tyrosine 332 to phenylalanine converts dopa de-carboxylase into a decarboxylation-dependent oxidative deaminase. J. Biol. Chem..

[36] Giardina G., Montioli R., Gianni S., Cellini B., Paiardini A., Voltattorni C.B., Cutruzzola F. (2011). Open conformation of human DOPA decarboxylase reveals the mechanism of PLP addition to Group II decarboxylases. Proc. Natl. Acad. Sci. USA.

[37] Montioli R., Dindo M., Giorgetti A., Piccoli S., Cellini B., Voltattorni C.B. (2014). A comprehensive picture of the mutations asso-ciated with aromatic amino acid decarboxylase deficiency: From molecular mechanisms to therapy implications. Hum. Mol. Genet..

[38] Montioli R., Bisello G., Dindo M., Rossignoli G., Voltattorni C.B., Bertoldi M. (2020). New variants of AADC deficiency expand the knowledge of enzymatic phenotypes. Arch. Biochem. Biophys..

[39] Montioli R., Paiardini A., Kurian M.A., Dindo M., Rossignoli G., Heales S.J., Pope S., Voltattorni C.B., Bertoldi M. (2016). The novel R347g pathogenic mutation of aromatic amino acid decarboxylase provides additional molecular insights into enzyme catalysis and deficiency. Biochim. Biophys. Acta Proteins Proteom..

[40] Montioli R., Janson G., Paiardini A., Bertoldi M., Voltattorni C.B. (2018). Heterozygosis in aromatic amino acid decarboxylase deficiency: Evidence for a positive interallelic complementation between R347Q and R358H mutations. IUBMB Life.

[41] Montioli R., Oppici E., Cellini B., Roncador A., Dindo M., Voltattorni C.B. (2013). S250F variant associated with aromatic amino acid decarboxylase deficiency: Molecular defects and intracellular rescue by pyridoxine. Hum. Mol. Genet..

[42] Montioli R., Battini R., Paiardini A., Tolve M., Bertoldi M., Carducci C., Leuzzi V., Voltattorni C.B. (2019). A novel compound heterozygous genotype associated with aromatic amino acid decarboxylase deficiency: Clinical aspects and biochemical studies. Mol. Genet. Metab..

